# Supplementary material for: Patterns of Intron Gain and Loss in Fungi
Source: PLoS Biol. 2004 Nov 30;2(12):e422. doi: 10.1371/journal.pbio.0020422 (PMC532390; doi:10.1371/journal.pbio.0020422)
Supplement: Table S1 — Also available at http://genes.mit.edu/NielsenEtAl/. (4.3 MB ZIP). [file pbio.0020422.st001.zip › NielsenEtAl/html/604.html]

AN7325.1.NCU01192.1.MG08071.1.FG07175.1


```
 CLUSTAL W (1.82) Multiple Sequence Alignments - Introns Inserted


Sequence 1: MG08071.1	1103 aa
Sequence 2: FG07175.1	1104 aa
Sequence 3: NCU01192.1	1104 aa
Sequence 4: AN7325.1	1044 aa
Alignment Length: 1114 aa
Number Identitical Residues: 677 aa
Alignment Score (without introns) 31335


MG08071.1 	-MPAAVLPQKREFGEASNARKNIS----ATPSSSKKRKIEPPASSPAASRFKSSQ---AN
NCU01192.1	-MAPSALPQKRAFGEASSARSNIHNLPAASSDSAKKRRLDDVLPS---SRFRTFKNDGKG
FG07175.1 	-MTSTIASHKRVLSEN-NARQNIT----STLASTKKRKVDAIPSSPAAARVPGSQNDHRS
AN7325.1  	MTEAIVMPQKRVLGDASNNPRGVVN----SPDTIKKRKIDMQNTVKPSPRAPNGQ----R
          	   .   .:** :.: :.   .: .    :  : ***:::   .  .:.*    :     

MG08071.1 	KLGSSQPKSAFESEVLEKLSQDISDAKHNNTEKDQSWSRPPIPADFSPKTSKLCFQSIEA
NCU01192.1	RIPSTQPKSAFESEVLERMSQDISDLKQNNSEKDQAWERPPLPATYNPATDSLCFQSIEA
FG07175.1 	KMASSQPKSAFESEVLEKLSQDLSDRKRNSTEKDQAWDRPPV-VDFVPERDSLCFQSIEA
AN7325.1  	KVGSSQQKSQFEEEVLEKLTQDISGLKENNSEKDQQWERPPL-GEFDPTKDNICFQQIDA
          	:: *:* ** **.****:::**:*. *.*.:**** *.***:   : *  ..:***.*:*

MG08071.1 	EEGTLHGGQATVKLFGVTE~DGNSVMLHVKEFRHYLYVAAPTSFGQKDCNAFKAFLETNI
NCU01192.1	EEGFLHGGQATIKLFGVTE~NGNSVLLHVKDFKHYLYVQAPVSFGPDDCPKYRAFLETQL
FG07175.1 	EEGTLHGGRATVKLFGVNE~AGNSVMLHVTDFKHYLYVPAPVNFQPQDCAAFKAYLETQV
AN7325.1  	EEGTTMG-KPAIRLFGVTE0AGQSVLLHVTGFQHYLYIAAPVGFTKEDCDPYRAFLESKI
          	***   * :.:::****.*  *:**:***. *:****: **..*  .**  ::*:**:::

MG08071.1 	AQHQPAIHSVQMTMRENIYGFQNNTQNPFLKITVTDPKFINKVRSTIES--GNANWKGMW
NCU01192.1	AMPTPAIHSVTLTMRENMYGFQGNTQNPYIKVTVNDPKFLPRVRRLIET--NKANWKGMW
FG07175.1 	AQHQPSIHSVAFAMRENIYGFQGNQSKPYLKVTVTDPKFINRVRTTIQS--GNANWKGMW
AN7325.1  	GQFPPVIQSVQITMRENIYGFQGNKKSYYLKITVTEPRHISKLRSALETKAQTVNYKGLW
          	.   * *:** ::****:****.* .. ::*:**.:*:.: ::*  :::.: ..*:**:*

MG08071.1 	K-HDGGIMTFDNIQYVLRFMVDCK0IQGMSWVEAPAATYTLVPESQRQSNCQIEAEMSYR
NCU01192.1	K-SDGSIMTFDDIQYLLRFMVDCS0IAGMSWVEAPAGSYQLVND--KHSNCQIEAEMSYR
FG07175.1 	R-NDGEVQTFDNLQYLLRFMVDCK0VRGMSWVEAPAKTYKLVNDHIRQSNCQIEAEVSYL
AN7325.1  	TNADGGILTFDNIQYLLRFMIDTD~IQGMSWVEAPAGKYELIAPQDKVSNCQLEARIDYR
          	 . ** : ***::**:****:* . : ********* .* *:    : ****:**.:.* 

MG08071.1 	DLIAHKPVGEWSKMAPLRILSFDIECAGRKGIFPEANMDPVIQIANVVTKYGDKKPFVRN
NCU01192.1	DLIAHKPVGEWSKMAPLRILSFDIECAGRKGIFPEPQHDSVIQIANIVTKYGEKKPFVRN
FG07175.1 	DLIAHEPVGEWSKMAPLRILSFDIECAGRKGIFPEANHDPVIQIANIVTKYGEKKPFVRN
AN7325.1  	SLISHAPVGDWAKMAPLRILSFDIECAGRKGIFPEPNHDPVIQIANVVTRYGESKPFIRN
          	.**:* ***:*:***********************.: *.******:**:**:.***:**

MG08071.1 	VFCLDQTSPIVATQILEFSQEQKMLSSWRNFMVEVDPDIITGYNIANFDFPYLLDRARHL
NCU01192.1	VFCLDTTSPIVATQILSFDREEDMLREWRNFLEQVDPDIIIGYNIANFDFPYLLDRAKHL
FG07175.1 	VFCLQETSSIVATQILAYEKEEKMLSDWQKFLIRTDPDIITGYNISNFDFPYLLDRAKHL
AN7325.1  	VFVMNTCSLIVNTQILEFQQEEKMLMAWRDFIQKVDPDVIIGYNIANFDFPYLLDRAKHL
          	** ::  * ** **** :.:*:.**  *:.*: ..***:* ****:***********:**

MG08071.1 	KVKDFDLWSRIPSIKSVAKESNFSSKQMGNRDTKATNINGRLQLDLLQLIQRDHQLRSYT
NCU01192.1	KVKDFDFWSRTR-VRSVAKETNFSSKQMGNRDTKATNTNGRLQLDMLQLVQRDYQLRSYT
FG07175.1 	KVAGFEYWTRIPSMQSRAKETNFSSKQMGNRDTKATNTNGRLQLDLLQLVQRDHQLRSYT
AN7325.1  	KCTNFPYWTRLNGMRSEAKDTNFSSKQMGNRDTKATNTNGRLQLDLLQLVQRDHNLRSYT
          	*  .*  *:*  .::* **::**************** *******:***:***::*****

MG08071.1 	LNSVCSHFLGEQKEDVHHTMITELFNGTPESRRRLALYCLKDAYLPQRLMDKLSCLENYT
NCU01192.1	LNSVCSHFLGEQKEDVHHSMITELFEGTPESRRRLALYCLKDAYLPQRLMDKLSCLENYT
FG07175.1 	LNSVCANFLGEQKEDVHHTMITELFNGTPESRRRLALYCLKDAYLPQRLMDKLSCLENYT
AN7325.1  	LNSVSYEFLGEQKEDVHHTMITELYNGTPDSRRRLAVYCLKDAYLPQRLMDKLMCLVNYT
          	****. .***********:*****::***:******:**************** ** ***

MG08071.1 	EMSRVTGAPFNFLLTRGQQVRFLSQLFRKALEQKLVIPNLSVQSSDEQYEGATVIEPTRG
NCU01192.1	EMARVTGVPFNFLLARGQQVKFLSQLFRKALEQKLVIPNLRPESSEEQYEGATVIEPTRG
FG07175.1 	EMARVTGVPFNFLLSRGQQVKFISQLFRKALEQKLVIPNLKSEASDEQYEGATVIEPTRG
AN7325.1  	EMARVTGVPFNFLLSRGQQVKFLSQLYRKALQQQLVIPNMKS-TDEQDYEGATVIEPIRD
          	**:****.******:*****:*:***:****:*:*****:   :.:::********* *.

MG08071.1 	YYDVPVATLDFASLYPSIMQAHNLCYTTLVTKKAIDAFGLKKDEDYIVTPNGDFFVTIKQ
NCU01192.1	YYDVPIATLDFASLYPSIMQAHNLCYTTLIKKRDIERWDLKKDEDYIVTPNGDMFVTTKQ
FG07175.1 	YYDVPIATLDFASLYPSIMQAHNLCYTTLVSKKAIEAFNLQKDEDYIVTPNGDTFVTIKQ
AN7325.1  	YYDVPIATLDFASLYPSIIQAHNLCYTTLLNKNSVAG--MKKDEDYIVTPNGDMFCTPKV
          	*****:************:**********:.*. :    ::************ * * * 

MG08071.1 	RKGLLAQILEELLAARKQAKRELAVETDPFKKAVLNGRQLALKISANSVYGLTGATNGKL
NCU01192.1	RKGLLAQILEELLSARKQAKRELAVEKDPFKKAVLNGRQLALKVSANSVYGLTGATNGKL
FG07175.1 	RKGLLAQILEELLSARKQAKRELAVETDPFKKAVLNGRQLALKISANSVYGLTGATTGKL
AN7325.1  	RKGLLSQILEELLSARKKAKKELAQETDPFKKAVLNGRQLALKVSANSVYGLTGATVGKL
          	*****:*******:***:**:*** *.****************:************ ***

MG08071.1 	PCLEIASSTTSFGRQMIEKTKEEVEKKYRVANGYSHDAQVIYGDTDSVMVKFGTKDLAEA
NCU01192.1	PCLEIASSVTSFGRQMIERTKKEVEERYTIANGYSHDAQVIYGDTDSVMVKFGTKDLAEA
FG07175.1 	PCLEIASSTTSFGRKMIEKTKEEVEGRYNIANGYSHDAQVIYGDTDSVMIKFGTKDLQEA
AN7325.1  	PCLAIASSTTSYGRQMIEKTKQEVEARYTIANGYSHDAKVIYGDTDSVMVKFGVTELEEA
          	*** ****.**:**:***:**:*** :* :********:**********:***..:* **

MG08071.1 	MKLGEEAANYVSSKFIKPIKLEFEKVYFPYLLINKKRYAGLYWTKPEKYDKMDTKGIETV
NCU01192.1	MKLGQDASEYVSGKFIKPIKLEFEKVYFPYLLINKKRYAGLYWTKPEKYDKMDTKGIETV
FG07175.1 	MKLGEEASKYVSSKFVKPIKLEFEKVYFPYLLINKKRYAGLYWTKPEKYDKMDTKGIETV
AN7325.1  	MKLGQEASEYVSSKFIKPIKLEFEKVYFPYLLINKKRYAGLYWTNPKKYDKMDTKGIETV
          	****::*::***.**:****************************:*:*************

MG08071.1 	RRDNCLLVQTVIEKVLRMILIDQDVPGAQE2YVKETIAELLQNKVDMSKLVITKALTKDD
NCU01192.1	RRDNCLLVQTVIEKVLRMILIDQDVPGAQA2YVKDTIADLLQNKIDMSKLVITKALTKEN
FG07175.1 	RRDNCLLVQTVIEKVLRMILIDQDVPGAQE2YVKETIADLLQNKVDMSKLVITKALTKDD
AN7325.1  	RRDNCLLVQNVIETVLHKILIDRDVEAAQE2YVKDTISDLLQNKIDMSKLVITKALTKDS
          	*********.***.**: ****:** .**  ***:**::*****:*************:.

MG08071.1 	YSAKQAHVELAQRMKKRDAGSAPGLGDRVAYVMIKGAT~GSKNFERSEDPIYVLENNVPI
NCU01192.1	YDAKQAHVELAQRMKKRDAGSAPGLGDRVAYVMVKGAT~GSKNFERSEDPIYVLEHNVPI
FG07175.1 	YAAKQAHVELAQRMKKRDAGSAPGLGDRVAYVMIRGPA~GAKNFEKSEDPIYVLEHNVPI
AN7325.1  	YTAKQAHVELAERMRKRDAGSAPTLGDRVAYVIVKGTA1---------------------
          	* *********:**:******** ********:::*.:                      

MG08071.1 	DTKYYLDNQLAKPLGRIFEPILGETKAKSLLMGDHTRVVSVAASSVGGLMKFAKKTQTCM
NCU01192.1	DTKYYLDNQLAKPLGRIFEPILGETKAKSLLHGDHTRVVSVAAPTVGGLMKFAKKTQTCM
FG07175.1 	DTRYYLDNQLAKPLGRIFEPILGETKARSLLTGDHTRVISVAAPTVGGLMKFAKKTQTCM
AN7325.1  	-----------------------------------HEVHRHSAPTMGGLMKFTKKTQTCL
          	                                    .*   :*.::******:******:

MG08071.1 	ACKKPLTGKEESQGAVCADDAPRVGELYKKTLDRVSDLEVRFGRLWTQCQRCQGSMHCEV
NCU01192.1	GCKKPLTGKEESQGAVCADDAPRVGELYKKTLDRVSDLEVRFGRLWTQCQRCQGSMHCEV
FG07175.1 	GCKKPLTGKEESEGAVCSNCAPRVGELYKKTLDRVSDLEVRFGRLWTQCQRCQGSMHCEV
AN7325.1  	GCKKPLTGKDVMAGAVCESCRPRLGELYTKSMNKMSDLEVRFGRLWTQCQRCQGSLHCEV
          	.********:   **** .  **:****.*:::::********************:****

MG08071.1 	ICSSKDCPIFYMRMKAKKDLEDGNKELQRFDFDRAAIW
NCU01192.1	ICSSKDCPIFYMRMKAKKDLEDANKELQRFDFDAAAMW
FG07175.1 	ICSSKDCPIFYMRMKAKKDLEDAGRELTRFDADQAAMW
AN7325.1  	ICSSRDCPIFYMRMKAKKDVEDAQKELARFDFDAGAW-
          	****:**************:**. :** *** * .*
```
